# Juvenile Myoclonic Epilepsy: Seizure and Social Outcomes in Taiwan

**DOI:** 10.3390/healthcare11081197

**Published:** 2023-04-21

**Authors:** Siew-Na Lim, Tony Wu, Wei-En Johnny Tseng, Chun-Wei Chang, Hsiang-Yao Hsieh, Mei-Yun Cheng, Hsing-I Chiang, Chih-Hong Lee, Wey-Ran Lin, Chun-Jing Liu

**Affiliations:** 1 Section of Epilepsy, Department of Neurology, Chang Gung Memorial Hospital at Linkou Medical Center, Taoyuan 333, Taiwancmrpg3c1121@cgmh.org.tw (C.-J.L.); 2 College of Medicine, Chang Gung University, Taoyuan 333, Taiwan; 3 PhD Program in Biomedical Engineering, Chang Gung University, Taoyuan 333, Taiwan; 4 Department of Gastroenterology and Hepatology, Chang Gung Memorial Hospital at Linkou Medical Center, Taoyuan 333, Taiwan

**Keywords:** juvenile myoclonic epilepsy, seizure, outcomes, social status, predictor

## Abstract

Patients with juvenile myoclonic epilepsy (JME) may not achieve seizure freedom despite optimal treatment with antiseizure medications (ASMs). The aim of this study was to investigate the clinical and social features of patients with JME, and to determine the factors associated with outcomes. We retrospectively identified 49 patients with JME (25 females, mean age 27.6 ± 8.9 years) who were assessed at the Epilepsy Centre of Linkou Chang Gung Memorial Hospital in Taiwan. The patients were divided into two groups, those who were seizure-free and those with ongoing seizures according to their seizure outcome at the last follow-up for one year. Clinical features and social status were compared between these two groups. Twenty-four (49%) of the JME patients were seizure-free for at least one year, while 51% continued to experience seizures despite being treated with multiple ASMs. The presence of epileptiform discharges in the last electroencephalogram and seizures during sleep were significantly associated with worse seizure outcomes (*p* < 0.05). The patients who were seizure-free had a higher employment rate compared to those who continued to experience seizures (75% vs. 32%, *p* = 0.004). Despite receiving ASM treatment, a considerable proportion of the patients with JME continued to have seizures. Moreover, poor seizure control was associated with a lower employment rate, which may lead to negative socioeconomic consequences related to JME.

## 1. Introduction

Juvenile myoclonic epilepsy (JME) is a subtype of idiopathic generalized epilepsy (IGE). JME is widely recognized, and accounts for 5–10% of all cases of epilepsy and 18–24% of IGE [1,2,3]. Although appropriate antiseizure medications (ASMs) can control JME, new evidence suggests that drug-resistant forms account for 10–35% of adult JME patients [4]. Predictive factors for JME and their correlation with treatment outcomes and disease evolution are being sought. However, the long-term seizure outcomes of JME are still controversial, and the factors that may predict seizure outcomes remain unclear.

Several clinical predictive factors have been reported to be associated with ASM resistance in JME, including the coexistence of myoclonic, absence, and generalized tonic-clonic seizures (GTCS) [4,5], as well as psychiatric comorbidities [6,7]. Although typical electroencephalogram (EEG) features of JME consist of generalized spike/poly-spike and waves, focal EEG anomalies are also evident in patients with JME. Some reports have suggested that these focal findings are associated with a higher risk of ASM resistance in JME patients [7,8], but other studies have failed to confirm this prognostic association [9,10]. Many studies have investigated the association between various factors, such as gender [11,12], age at seizure onset [13,14], seizure types [15,16,17], history of febrile seizures [14], family history of epilepsy [7,12], and delayed diagnosis [18], with this drug-resistant JME; however, the results remain inconsistent.

The long-term social outcomes for JME patients are also an important issue, with conflicting reports on the social consequences of having JME [3,19,20]. Although case series of JME patients have been reported from various countries, including India [18,21] and China [14,17], clinical studies in Asia are relatively rare. As JME has a strong genetic predisposition, additional research from diverse nations and cultures is necessary to better understand the condition. Therefore, this study aimed to identify correlations between clinical characteristics and prognosis in patients with JME, and to investigate the social outcomes of these patients in Taiwan.

## 2. Materials and Methods

### 2.1. Subjects

This retrospective study was conducted at the Epilepsy Center of Linkou Chang Gung Memorial Hospital, a tertiary epilepsy center in Taiwan. We included consecutive patients with JME who were diagnosed by a qualified epileptologist. The diagnosis of JME was based on Class II criteria as proposed by a consensus amongst international experts on JME [22]. These criteria encompassed the following: (1) myoclonic jerks predominantly occurring on awakening; (2) myoclonic jerks facilitated by sleep deprivation and stress and provoked by visual stimuli and praxis or GTCSs preceded by myoclonic jerks; (3) EEG showing a normal background and at least one interictal generalized spike or poly-spike and waves with some asymmetry allowed with or without myoclonic jerks; (4) no mental retardation or deterioration; and (5) age at onset of between 6 and 25 years. The inclusion criteria were patients: (1) with a diagnosis of JME as defined above; (2) with at least one year of follow-up after the first ASM prescription; and (3) who had complete information with respect to clinical details, EEG and imaging reports, and treatment history. The exclusion criteria were as follows: (1) the presence of focal neurological or intellectual impairment; (2) focal seizures; (3) abnormal brain imaging; (4) progressive neurologic dysfunction; and (5) poor drug adherence. This study was approved by the Institutional Review Board of Linkou Chang Gung Memorial Hospital.

### 2.2. Collection of Demographic and Clinical Data

Demographic and clinical data were collected through a detailed review of individual medical records. The collected data included age, gender, age at epilepsy onset, duration of epilepsy, history of febrile seizure, family history of epilepsy, ASM history, EEG features, and results of physical and brain imaging examination. The details of seizures, including seizure types, age at onset of each seizure type, occurrence of seizures throughout wakefulness or sleep, seizure-provoking factors, and seizure outcomes were recorded. Social aspects such as education level, marital status, employment status, and transportation method at the time of last follow-up were also recorded.

The response to ASMs was evaluated according to the latest medical record following the commonly accepted criteria proposed by the International League Against Epilepsy (ILAE) task force [23]. The patients were categorized into two groups: those who were seizure-free and those with ongoing seizures based on their seizure control in the past one year. To be classified as seizure-free, a patient had to have been free of seizures (of any type) for at least 12 months at the last follow-up. Patients who had experienced seizures in the past one year were considered to have ongoing seizures, even if the seizures occurred due to external factors such as sleep deprivation; this is in line with the criteria proposed by the ILAE task force [23]. Patients in whom nonadherence to ASMs was documented or suspected were excluded to prevent the inclusion of pseudo-refractory cases.

### 2.3. Statistical Analysis

Data were analyzed using SPSS version 21 (SPSS, Chicago, IL, USA). Descriptive statistics were used to summarize baseline characteristics. Categorical data have been presented as numbers and percentages, and continuous data are described as mean ± standard deviation. Fisher’s exact test was used to compare categorical dependent variables between the seizure-free and ongoing seizure groups. The Mann–Whitney U test was used to compare continuous variables between the two outcome groups after testing for normality using the Kolmogorov–Smirnov test. Multivariate logistic regression analysis was further carried out to determine the predictors of seizure outcomes. Odds ratios and 95% confidence intervals were calculated. Results were considered significant at a *p*-value ≤ 0.05.

## 3. Results

### 3.1. Clinical Characteristics of the Patients with JME

A total of 49 JME patients (25 females, mean age 27.6 ± 8.9 years) were included in the study, and their clinical characteristics are summarized in Table 1. The average age at the onset of the first clinical manifestation was 14.2 ± 3.1 years, while the average age at the diagnosis of epilepsy was 16.2 ± 5.4 years. The diagnosis of JME was delayed until 21.1 ± 8.7 years. The average duration of epilepsy was 13.4 ± 9.4 years in the overall cohort of patients. Of the 49 patients, 7 (14.3%) had a positive history of epilepsy, and 6 (12.2%) had a history of febrile seizures.

In terms of the type of seizures, 18 patients (36.7%) had myoclonic seizures as their first clinical manifestation, while 26 patients (53.1%) had GTCS, and only 5 patients (10.2%) showed absence seizures initially. Among the whole study population, 25 (51%) had a history of both myoclonic and GTCS, 2 patients (4.1%) had both myoclonic and absence seizures, 16 patients (32.7%) had absence seizures in addition to myoclonic and GTCS, and the remaining 6 patients (12.2%) had myoclonic seizures only.

The timing of seizures varied among the patients. Twenty-two patients (44.9%) only had seizures on awakening, while 14 patients (28.6%) had seizures only during sleep. Twelve patients (26.5%) had seizures both while awake and asleep. Sleep deprivation was reported as a seizure precipitant by 10 patients (20.4%), flicking lights by 8 patients (16.3%), and stress by 7 patients (14.3%).

All of the patients except one were treated with ASMs. The average number of ASMs used at the last follow-up was 1.6 ± 0.9 (range 0 to 4). Twenty-seven patients were on monotherapy, and 21 were on polytherapy. The most commonly used ASMs were levetiracetam (69.4%), valproic acid (34.7%), and clonazepam (28.6%). In total, 34 patients (69.4%) received levetiracetam, with 19 of them being on monotherapy; 17 patients (34.7%) received valproic acid, with 8 of them being on monotherapy. The average number of ASMs tried during the entire course of treatment was 3.0 ± 1.9.

### 3.2. Seizure Outcomes of the Patients with JME

All of the patients were followed up for at least one year at the epilepsy clinic. Of the 49 patients, 24 (49%) were seizure-free during the past 12 months, while 25 (51%) continued to experience seizures despite ASM treatment. The analysis of the clinical variables assessed as being potential predictors of one-year seizure outcomes showed that seizures during sleep and epileptiform discharges in the last EEG were significantly associated with ongoing seizures (*p* < 0.05). No other clinical variables were significantly different between the two groups with regards to one-year seizure control (Table 2).

### 3.3. Predictors of Seizure Outcomes

Multivariate logistic regression analysis revealed that the occurrence of seizures during sleep and the presence of epileptiform discharges on the most recent EEG were significant predictors of ongoing seizures (Table 3).

### 3.4. Social Outcomes of the Patients with JME

Of the 49 JME patients, 13 (26.5%) were married and 28 (57.1%) reported having completed college education. Regarding modes of transportation, nearly half of the patients (49.0%) reported using public transportation as a means of travel in their daily routine. In contrast, the remaining 25 patients reported operating motor vehicles, specifically cars or motorbikes, or both.

**Figure 1:**
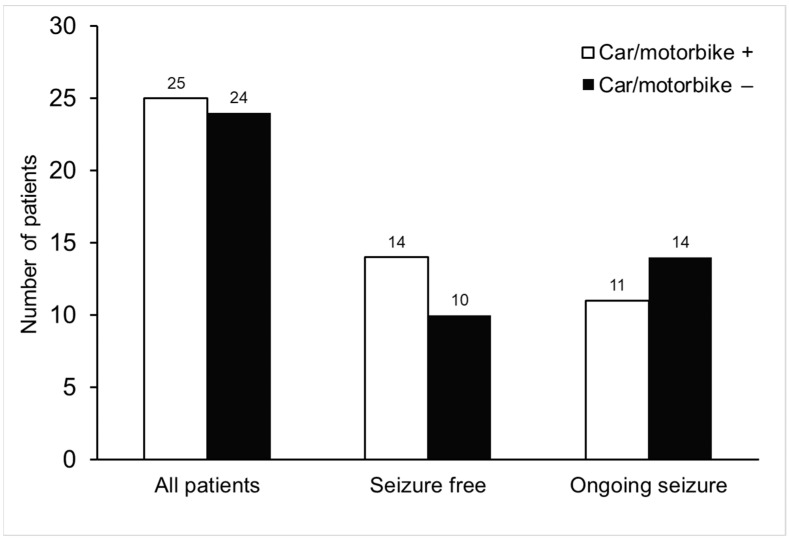
The frequency of vehicle operation among patients who were seizure-free and those with ongoing seizures. The numbers of patients who drove cars or motorbikes and those who did not operate any vehicles are presented for each subgroup. No statistically significant differences were observed between the groups of vehicle operators and non-operators in both seizure-free and ongoing seizure subgroups (*p* = 0.193).

Table 4 provides an overview of the social outcomes of the seizure-free and ongoing seizure groups. A greater proportion of seizure-free patients were employed compared to those with ongoing seizures (*p* < 0.05). The regular use of a car or motorbike was reported by 14 patients (58.3%) who were seizure-free, and by 11 patients (22.4%) with ongoing seizures. We further compared the number of patients who operated vehicles against those who did not. The results demonstrated that there were no significant differences between the groups of vehicle operators and non-operators in both the seizure-free and ongoing seizure groups (Figure 1, *p* = 0.193).

## 4. Discussion

In this study, we analyzed the clinical characteristics, seizures, and social outcomes of Taiwanese patients with JME. Nearly half of the patients remained seizure-free for one year, and worse seizure outcomes were associated with seizures during sleep and the presence of epileptiform discharges during the last EEG. The patients who were seizure-free had better social status in terms of employment when compared to those who continued to experience seizures.

Various studies have reported differing rates of seizure freedom in patients with JME. A retrospective study of 66 patients reported that 59.1% of patients remained seizure-free for at least 5 years [15]. Another recent study involving 61 patients diagnosed with JME for more than 15 years reported that 65.5% of the patients experienced terminal remission lasting for 5 years [13], while 81.4% of the patients with newly diagnosed epilepsy achieved remission of all seizure types lasting for at least 2 years [24]. Despite being treated with appropriate and adequate ASMs, more than half (51%) of the patients in our study did not achieve seizure freedom. Our hospital is a tertiary epilepsy center and typically treats patients with more severe or refractory forms of epilepsy than those at secondary centers, which could explain the high percentage of ongoing seizures. Furthermore, we defined ongoing seizures as encompassing the persistence of all seizure types. Patients with occasional myoclonic seizures may not want to take higher doses of medications, leading to continued seizures. Similarly, Asadi-Pooya et al. reported that only 39.3% of patients with JME experienced a seizure-free period of at least 12 months for all seizure types. However, with regard to the most debilitating type of seizure (i.e., GTC), over two-thirds of their patients with JME may achieve seizure-free status during follow-up [20]. In addition, a meta-analysis of 43 relevant studies concluded that 35% (with a 95% confidence interval of 29–41%) of patients with JME had drug-resistant seizures. The rates of refractoriness were similar when evaluating 1-, 2-, and 3-year seizure-freedom rates, indicating that patients who are seizure-free for at least 1 year are likely to remain so [4]. A retrospective study conducted in South India revealed that seizures were controlled (<1 GTC per year) in 31%, 38%, and 60% of JME patients at the end of 1 year, 5 years, and 10 years of epilepsy, respectively, suggesting that early seizure control (within the first 5 years of epilepsy) resulted in better outcomes at the end of 10 years of epilepsy [21]. In contrast, another study reported rates of seizure freedom after 1, 3, and 5 years of ASM treatment initiation of 64.8%, 29.5%, and 14.6% in JME patients, respectively [17]. These varying rates of seizure-free status could be due to the different methodologies and variables related to outcomes not being consistent in various studies, as well as the sample sizes, all of which limits comparisons. Nevertheless, taken together, the results of these studies challenge the notion that JME is a benign epilepsy, and physicians must be careful when counseling JME patients about the prognosis. The seizure outcome data from previous studies in the last 10 years (2013–2023) are presented in Table 5.

It is essential to identify patients with drug-resistant JME promptly and manage them successfully, as refractory seizures can lead to progressive brain damage, severe cognitive and psychiatric consequences, socio-economic burden [29], and a higher risk of mortality [30]. As mentioned above, various predictors have been suggested; however, data for many of them have not been corroborated by subsequent studies. In the present study, we found that the patients who only had seizures during sleep and the presence of epileptiform discharges in the last EEG were significantly associated with worse seizure outcomes, which has not been reported before. However, the limited number of patients should be taken into account when interpreting the results.

The social outcomes of patients with epilepsy are strongly linked to the status of their seizure control. For instance, individuals with drug-resistant epilepsy who experience a decrease in seizures following epilepsy surgery have been shown to have a higher chance of achieving social milestones, such as gaining a driver’s license, securing employment, and establishing long-term relationships, in comparison to those who continue to experience seizures [31]. A study conducted in Germany found that early and long-term seizure control could enhance the long-term social outcomes in patients with JME [25]. Of the patients in our study, 30.6% were students and not part of the workforce, while 76.5% of the remaining 34 patients were employed, which is lower than the employment rate in Taiwan as a whole, which was 96.3% in April 2019 [32]. Similar studies in Denmark and Iran also reported lower employment rates in JME patients than in the general population [3,20]. Notably, we found a significantly higher employment rate in the patients who were seizure-free compared to those who continued to experience seizures. This is likely due to the unpredictable nature of seizures, which can have a serious impact on one’s ability to work. In addition, the fear of having a seizure at work often limits the ability of individuals with epilepsy to work to their full potential [33]. While previous studies generally agree that patients with more frequent seizures face a greater burden and higher rates of unemployment, some researchers have argued that the evidence so far does not definitively establish a direct relationship between seizure frequency and employment status. A systematic review conducted by Wo and colleagues found that individuals with uncontrolled seizures had similar employment rates to those with controlled seizures, suggesting that other factors may contribute to the employment status of people with epilepsy [34]. These factors are multifactorial and may include adverse side effects of ASMs, stigma associated with epilepsy, and psychosocial variables such as self-esteem, coping style, and self-efficacy. In addition, patients with epilepsy may face challenges in securing or maintaining a job due to legal restrictions on driving, limited access to public transportation, and difficulties in negotiating workplace accommodation [35]. However, we did not investigate the details of these variables, and therefore we could not determine the specific causes of the observed association between employment status and seizure control, and so further research is needed to fully understand this complex relationship.

The marriage rate in our JME patients was 26.5%, which is lower than the marriage rate of 49.9% reported in the general population of Taiwan in 2021 [36]. However, the marital status of JME patients did not differ significantly between those with ongoing seizures and those who were seizure-free. A previous study conducted in Denmark reported that JME patients had a lower rate of marriage than controls [3], while a study in Iran found that the rate of marriage among JME patients was similar to that of the general population [20]. Nonetheless, it has also been reported that people with epilepsy are more likely to remain unmarried than those without epilepsy [37]. This may be attributed to a negative attitude toward people with epilepsy with regard to social relationships, which can be more stressful than epilepsy itself, and lead to social isolation, thereby limiting the opportunities for social interaction, including marriage [38]. In addition, an awareness survey conducted in Taiwan revealed that 72% of respondents objected to their children marrying a person with epilepsy [39], which could also contribute to the low marital rate observed in the JME patients in our study. All patients in this study were literate and received education beyond junior high school, with 49.0% having a college education, consistent with the general population of Taiwan [40].

We also found that 51% of the patients with JME reported driving a car or motorbike in their daily lives, similar to a previous report [20]. In Taiwan, individuals with epilepsy or seizures must disclose their condition when applying for a driver’s license and they can only obtain a license for a car or motorbike after being seizure-free for two years, as confirmed by their physician. The seizure-free period required to obtain a driving license varies between countries, and many people with drug-resistant epilepsy still drive despite the risk [41]. In the current study, 11 of the patients with ongoing seizures reported that they were still driving. Although driving restrictions may be necessary to ensure safety, it should be noted that being restricted from driving due to seizures can have a significant impact on a person’s independence, employment, and quality of life [28]. Thus, it is important to evaluate each patient’s driving situation individually and provide advice that maximizes safety for both the patient and public [28].

There are several limitations to this study that should be acknowledged. First, its observational design, relatively small sample size and reliance on medical records for data retrieval may have introduced bias, and may limit the generalizability of the findings. Second, the study was limited to a hospital-based sample and did not include a comparison group of individuals without epilepsy, precluding a full understanding of the relationship between epilepsy and patients’ social outcomes within the broader community. Future studies should aim to address this limitation by conducting population-based studies with appropriate control groups. Lastly, the study was conducted at a single tertiary center, and therefore it may not represent the full spectrum of patients with JME, as individuals with milder forms of the disease may not have been referred to a tertiary hospital, leading to potential selection bias. Thus, well-designed prospective follow-up studies involving larger multicenter samples are necessary to draw more conclusive findings.

## 5. Conclusions

In summary, our findings show that more than half (51%) of the enrolled patients with JME continued to experience seizures despite treatment with multiple ASMs, suggesting that JME may not be as benign as previous studies have suggested. We identified specific predictors of poor seizure outcomes, including seizures during sleep and the presence of epileptiform discharges in the last EEG. Poor seizure control was associated with a lower employment rate, highlighting the potential negative socioeconomic consequences of drug-resistant JME, which is consistent with observations in studies of other types of epilepsy.

## Figures and Tables

**Table 1:** Characteristic of patients diagnosed with JME.

	JME Patients *n* = 49 (%)
**Age**, y, mean ± SD	27.6 ± 8.9
**Gender**, (Female: Male)	25: 24
**Family history of epilepsy**	7 (14.3)
**History of febrile seizures**	6 (12.2)
**Age of first seizure**, y, mean ± SD	14.2 ± 3.1
**Seizure duration**, y, mean ± SD	13.4 ± 9.4
**Age of diagnosis of epilepsy**, y, mean ± SD	16.2 ± 5.4
**Age of diagnosis of JME**, y, mean ± SD	21.1 ± 8.7
**Duration of follow up**, y, mean ± SD	11.4 ± 9.3
**Year of delayed diagnosis of JME**, y, mean ± SD	6.9 ± 9.1
**Seizure types**	
Myoclonic seizures only	6 (12.2)
Myoclonic + absence seizures	2 (4.1)
Myoclonic seizures + GTCS	25 (51.0)
Myoclonic + absence seizures + GTCS	16 (32.7)
**First clinical manifestation**	
Myoclonic seizures	18 (36.7)
GTCS	26 (53.1)
Absence seizures	5 (10.2)
**Timing of seizures**	
Awake only	22 (44.9)
Sleep only	14 (28.6)
Both awake and sleep	13 (26.5)
**Seizure precipitant factors**	
Sleep deprivation	10 (20.4)
Flicking light	8 (16.3)
Soundsensitive	3 (6.1)
Stress	7 (14.3)
Fatigue	2 (4.1)
Common cold	2 (4.1)
Alcohol intake	1 (2.0)
Exercise	1 (2.0)
**EEG features**	
Focal EEG discharges	4 (8.2)
Epileptiform discharges in the last EEG	30 (61.2)
**No. of ASM tried**, mean ± SD **Current ASM treatment** (range, 0–4)	3.0 ± 1.9 (range, 1–10)
No ASM	1 (2.0)
Monotherapy	27 (55.1)
Two ASMs	14 (28.6)
Three ASMs	4 (8.2)
Four ASMs	3 (6.1)
**Most common ASM type**	
Levetiracetam	34 (69.4)
Valproic acid	17 (34.7)
Clonazepam	14 (28.6)
**Seizure-free duration**	
>1 year	24 (49.0)
>6 months	27 (55.1)
>3 months	30 (61.2)

JME, juvenile myoclonic epilepsy; GTCS, generalized tonic-clonic seizures; EEG, electroencephalogram; ASM, antiseizure medication.

**Table 2:** Clinical factors distinguishing patients with and without seizure control for at least one year.

	Seizure Outcomes		
	Seizure-Free *n* = 24 (%)	Ongoing Seizures *n* = 25 (%)	*p*-Value
**Age**, y, mean ± SD	27.3 ± 7.4	27.9 ± 10.2	0.849 ^a^
**Gender**, Female	14 (58.3)	11 (44.0)	0.396 ^b^
**Family history of epilepsy**	4 (16. 7)	3 (12.0)	0.702 ^b^
**History of febrile seizure**	1 (4.2)	5 (20.0)	0.189 ^b^
**Age of first seizures**, y, mean ± SD	14.8 ± 3.0	13.7 ± 3.2	0.437 ^a^
**Seizure duration**, y, mean ± SD	12.5 ± 7.2	14.2 ± 11.2.	0.528 ^a^
**Age of diagnosis of epilepsy**, y, mean ± SD	16.3 ± 4.4	16.1 ± 6.3	0.825 ^a^
**Age of diagnosis of JME**, y, mean ± SD	19.6 ± 6.5	22.6 ± 10.3	0.458 ^a^
**Year of delayed diagnosis of JME**, y, mean ± SD	4.8 ± 5.0	8.8 ± 11.5	0.567 ^a^
**Duration of follow up**, y, mean ± SD	11.0 ± 7.9	11.8 ± 10.6	0.896 ^a^
**Seizure types**			
Myoclonic seizures only	4 (16.7)	2 (8.0)	0.417 ^b^
Myoclonic + absence seizures	0	2 (8.0)	0.490 ^b^
Myoclonic seizures + GTCS	15 (62.5)	10 (40.0)	0.156 ^b^
Myoclonic + absence seizures + GTCS	5 (20.8)	11 (44.0)	0.128 ^b^
**First clinical manifestation**			
Myoclonic seizure	10 (41.7)	8 (32.0)	0.551 ^b^
GTCS	13 (54.2)	13 (52.0)	1.000 ^b^
Absence seizure	1 (4.2)	4 (16.0)	0.349 ^b^
**Timing of seizures**			
Awake only	13 (54.2)	9 (36.0)	0.256 ^b^
Sleep only	3 (12.5)	11 (44.0)	0.025 ^b^ *
Both awake and sleep	8 (33.3)	5 (20.0)	0.345 ^b^
**Seizure precipitant factors**			
Sleep deprivation	4 (16.7)	6 (24.0)	0.725 ^b^
Flicking light	6 (25.0)	2 (8.0)	0.138 ^b^
Sound sensitive	0 (0)	3 (12.0)	0.235 ^b^
Stress	3 (12.5)	4 (16.0)	1.000 ^b^
Fatigue	2 (8.3)	0 (0)	0.235 ^b^
Common cold	0 (0)	2 (8.0)	1.000 ^b^
Alcohol intake	1 (4.2)	0 (0)	1.000 ^b^
Exercise	0 (0)	1 (4.0)	1.000b
**EEG features**			
Focal EEG discharges	2 (8.3)	2 (8.0)	1.000 ^b^
Epileptiform discharges in the last EEG	10 (41.7)	20 (80.0)	0.009 ^b^ *

JME, juvenile myoclonic epilepsy; GTCS, generalized tonic-clonic seizures; EEG, electroencephalogram. ^a^ Mann–Whitney U test, ^b^ Fisher’s exact test. * The presence of seizures during sleep and epileptiform discharges on the last EEG were significantly associated with ongoing seizures.

**Table 3:** Factors independently associated with ongoing seizures in the patients with JME.

	*p*	OR	95% CI for OR
**Attack during sleep**	0.018	0.136	0.026–0.711
**Epileptiform discharges in the last EEG**	0.008	0.140	0.033–0.597

JME, juvenile myoclonic epilepsy; EEG, electroencephalogram; OR, odds ratio; CI, confidence interval.

**Table 4:** Social outcomes’ association with seizure control in the patients with JME.

		Seizure Outcomes		
	All Patients *n* = 49 (%)	Seizure-Free *n* = 24 (%)	Ongoing Seizures *n* =25 (%)	*p*-Value
**Married**	13 (26.5)	6 (25.0)	7 (28.0)	1.000 ^b^
**Education**, y, mean ± SD	14.3 ± 2.9	14.5 ± 2.9	14.0 ± 2.9	0.480 ^a^
**College Education**	28 (57.1)	14 (58.3)	14 (56.0)	1.000 ^b^
**Employment**				
Employed	26 (53.1)	18 (75.0)	8 (32.0)	0.004 ^b^ *
Unemployed	8 (16.3)	1 (4.2)	7(28.0)	0.049 ^b^ *
**Student** (outside the labour force)	15 (30.6)	5 (20.8)	10 (40.0)	0.217 ^b^
**Transportation**				
Public transportation	24 (49.0)	10 (41.7)	14 (56.0)	0.396 ^b^
Motorbike only	12 (24.5)	5 (20.8)	7 (28.0)	0.742 ^b^
Car only	4 (8.2)	4 (16.7)	0 (0)	0.050 ^b^
Motobike or car	25 (51.0)	14 (58.3)	11 (44.0)	0.396 ^b^

JME, juvenile myoclonic epilepsy. ^a^ Mann–Whitney U test, ^b^ Fisher’s exact test. * Patients who were seizure-free had better employment outcomes compared to those with ongoing seizures.

**Table 5:** Seizure outcome studies of JME in the last 10 years (2013–2023).

	Study Design	Country (City) of Study	No of Pts, *n*	Age, year, Mean ± SD/ Mean (Range)	Female, *n* (%)	Follow Up, year Mean ± SD/ Mean (Range)	Seizure-Free, %	Ongoing Seizures, %
Senf et al. 2013 [15]	Single center	Germany (Berlin)	66	58.9 ± 13.8	33 (50)	44.6 ± 13.7	5 year: 59.1	40.9
Schneider-von Podewils. 2014 [25]	Single center	Germany (Greifswald)	33	52.3 ± 12.3	21 (63.3)	37.8 ± 13.9	>6 year: 54.5	45.5
Syvertsen et al., 2014 [16]	Single center	Norway (Trondheim)	40	Median (range) 47 (35–81)	25 (60) *	>20	5 year: 53 10 year: 33	47
Höfler et al., 2014 [5]	Single center	Austria (Salzburg)	175	Median (range) 38 (14–87)	111 (63.4)	Median (range) 8 (2–38)	1 year: 62 2 year: 53 >5 year: 15	38
Desai et al., 2016 [18]	Single center	India (Gujarat)	73	27 (6–57)	40 (54.8)	—	2 year: 84.9	15.1
Chowdhury et al., 2016 [6]	Single center	UK (Glasgow)	186	Median (range) 14 (2–32)	108 (58)	Median (range) 14 (2–32)	1 year: 92	8
Cação et al., 2018 [26]	Multi-center	UK (London, Chalfont)	240	38 ± 11.7	146 (52.1)	21.8 (1–60)	1 year: 47.5	50.4
Uchida et al., 2018 [27]	Single center	Brazil (São Paulo)	42	28.1 ± 11.1 ^#^ 32.9 ± 10.7	30 (71.4)	8.2 ± 5.0	31 ^§^	69
Zhang et al., 2019 [17]	Single center	China (Chengdu)	105	21.5 ± 5.3	57 (54.3)	47.9 ± 7.2	1 year: 64.8 3 year: 29.5 5 year: 14.6	35.2
Chen et al., 2020 [28]	Single center	China (Xiamen)	63	20.4 ± 6.9	30 (47.6)	—	5 year: 63.5	36.5
Viswanathan et al., 2021 [21]	Single center	India (Karnataka)	56	14.5 ± 5.1	30 (53.6)	10 year	10 year: 60.7 ^@^	39.3
Pietrafusa et al., 2021 [13]	Single center	Italy (Apulia)	61	37.6 ± 12.3	61 (65.6)	28.9 ± 8.2	5 year: 65.5	34.5
Asadi-Pooya et al., 2022 [20]	Single center	Iran (Shiraz)	135	35 ± 8	92 (68.1)	11 ± 2	1 year: 39.3	60.7
Cerulli Irelli et al., 2022 [24]	Single center	Italy (Rome)	113	Median (IOR) 64 (17–91)	78 (69)	Median (IOR) 17 (9.5–27)	2 year: 81.4 4 year: 67.3	18.6
Current study	Single center	Taiwan (Taoyuan)	49	27.6 ± 8.9	51 (25)	11.4 ± 9.3	1 year: 49	51

* This study enrolled 42 patients with JME, and 25 (60%) of them were female. A subset of 40 underwent seizure outcome analysis. ^#^ The average ages (± SD) of JME patients with or without eye-closure sensitivity were 28.1 ± 11.1 and 32.9 ± 10.7, respectively. ^§^ The definition of seizure-free period was not reported. ^@^ Patients were considered to have controlled seizures when they had <1 GTCS/year and no/very rare non-disabling myoclonia at the end of 10 years duration of epilepsy. —: not explicitly reported. JME (juvenile myoclonic epilepsy); pts, patients; y, year; SD, standard deviation; IOR, interquartile range.

## Data Availability

The datasets used and/or analyzed during the current study are available from the corresponding author on reasonable request.
